# Limitations and Promise of Retinal Tissue From Human Pluripotent Stem Cells for Developing Therapies of Blindness

**DOI:** 10.3389/fncel.2020.00179

**Published:** 2020-09-10

**Authors:** Ratnesh K. Singh, Igor O. Nasonkin

**Affiliations:** Lineage Cell Therapeutics, Alameda, CA, United States

**Keywords:** retinal organoids, disease modeling, pluripotent stem cells, retinal degeneration, photoreceptors, assembloids, drug screening, retinal pigment epithelium

## Abstract

The self-formation of retinal tissue from pluripotent stem cells generated a tremendous promise for developing new therapies of retinal degenerative diseases, which previously seemed unattainable. Together with use of induced pluripotent stem cells or/and CRISPR-based recombineering the retinal organoid technology provided an avenue for developing models of human retinal degenerative diseases “in a dish” for studying the pathology, delineating the mechanisms and also establishing a platform for large-scale drug screening. At the same time, retinal organoids, highly resembling developing human fetal retinal tissue, are viewed as source of multipotential retinal progenitors, young photoreceptors and just the whole retinal tissue, which may be transplanted into the subretinal space with a goal of replacing patient’s degenerated retina with a new retinal “patch.” Both approaches (transplantation and modeling/drug screening) were projected when Yoshiki Sasai demonstrated the feasibility of deriving mammalian retinal tissue from pluripotent stem cells, and generated a lot of excitement. With further work and testing of both approaches *in vitro* and *in vivo*, a major implicit limitation has become apparent pretty quickly: the absence of the uniform layer of Retinal Pigment Epithelium (RPE) cells, which is normally present in mammalian retina, surrounds photoreceptor layer and develops and matures first. The RPE layer polarize into apical and basal sides during development and establish microvilli on the apical side, interacting with photoreceptors, nurturing photoreceptor outer segments and participating in the visual cycle by recycling 11-trans retinal (bleached pigment) back to 11-cis retinal. Retinal organoids, however, either do not have RPE layer or carry patches of RPE mostly on one side, thus directly exposing most photoreceptors in the developing organoids to neural medium. Recreation of the critical retinal niche between the apical RPE and photoreceptors, where many retinal disease mechanisms originate, is so far unattainable, imposes clear limitations on both modeling/drug screening and transplantation approaches and is a focus of investigation in many labs. Here we dissect different retinal degenerative diseases and analyze how and where retinal organoid technology can contribute the most to developing therapies even with a current limitation and absence of long and functional outer segments, supported by RPE.

## Introduction

Retina is a great model for developmental neuroscience and a very attractive therapeutic target for biotech companies working in the field of regenerative medicine. There are only several types of retinal neurons (rod and cone photoreceptors, amacrine, horizontal, rod & cone bipolar and retinal ganglion cells), one type of glial cells (Muller glia) and a pigmented layer of supportive cells (Retinal pigment epithelium), which form the retina and help to carry out visual function (Wallace, 2011). On the contrary, the cortical organization in the brain is much more complex and has six layers of cortical neurons, each carrying different cell types with different function (Molyneaux et al., 2007; Lodato and Arlotta, 2015). This relative simplicity creates a promise for ease of recapitulation of this process in a dish (compared to brain), as well as (expected) relative ease of cell replacement therapies (again, compared to the brain). This, in turn, is very attractive to regenerative medicine and biotechnology, which aim to convert the already “understood” and “worked out” knowledge and concepts into robust technologies and therapies to transition science from the bench to patients. Age related macular degeneration (AMD), glaucoma and retinitis pigmentosa (RP) are the major retinal degenerative diseases affecting people worldwide. Understanding the causes and mechanisms of these diseases (outlined below) is a key for developing organoid-based *in vitro* models of these diseases for drug screening and disease modeling.

According to eye health data and statistics, summarized on NEI’s web site^1^ and in a recent study published by Varma et al. (2016), the number of people with most common eye diseases is going to double by 2050. *AMD* is a leading cause of vision loss in United States and mainly affects the central vision. According to statistics presented by Brightfocus foundation^2^ about 11 million of Americans have visual problem associated with AMD symptoms, and this number is projected only to increase and reach 22 million by 2050. The total number of people with macular degeneration worldwide is projected to be 196 million by now (2020) and 288 million by year 2040. About 30% of people age 75 and above have vision problems associated with AMD symptoms. Macular degeneration triggers loss of central vision and death of photoreceptors in the macula (*maculae*) (Molday, 1998; Molday and Moritz, 2015). *The dry form of AMD* accounts for 85 to 90 percent of all AMD cases (Klein et al., 1992; Bird et al., 1995; Vingerling et al., 1995). In dry AMD disruption and death of RPE causes accrual of yellow deposit (drusen) in the macula that contributes to accumulation of complement component and acute phase proteins leading to proinflammatory macrophage response (Ding et al., 2009) and eventually photoreceptor cell death. Geographic atrophy (GA) is devastating complication of dry AMD and is considered the late stage of this disease affecting more than 5 million people worldwide including nearly 1 million in the United States^4^ (Bird et al., 1995; Wong et al., 2014) (Friedman et al., 2004; Rudnicka et al., 2015. Geographic atrophy is a frequent cause of legal blindness (42% of patients with GA) (Klein et al., 1995) and severe (≥ 6 lines) vision loss (Sunness et al., 1999). Transplantation of human pluripotent stem cell (hPSC) derived-RPE into the subretinal space is one experimental therapy (in clinical trials now), which may address this condition (Schwartz et al., 2012, 2015, 2016; McGill et al., 2017; Cuzzani, 2018) and is aimed to support photoreceptors and prevent their cell death. In *wet (also neovascular or exudative) AMD* the abnormal growth of blood vessels (also known as choroidal neovascularization, CNV) beneath the macula causes separation between photoreceptors and RPE (Yeo et al., 2019). This is the only blinding disease, which has a robust treatment via suppressing neovasculogenesis with anti-Vascular Endothelial Growth Factor (VEGF) therapies (Meadows and Hurwitz, 2012) such as antibodies (or antibody fragments) to (bevacizumab, ranibizumab) (Rosenfeld et al., 2006; Raftery et al., 2007), VEGF-A soluble decoy (aflibercept) (Sarwar et al., 2016) or/and small molecules suppressing the tyrosine kinases induced by VEGF binding (lapatinib, sunitinib, pazopanib and a few other compounds). *Glaucoma* is another leading cause of irreversible vision loss. From 2011 to 2050, the number of people in the U.S. with glaucoma is expected to increase from 2.71 million in year 2011 to 3.72 million in year 2020 to 7.32 million by year 2050 (Vajaranant et al., 2012). Glaucoma affects retinal ganglion cells, carrying the visual signals from retina to brain, It is caused (mostly) by elevated intraocular pressure followed by loss of retinal ganglion cells and their axons (Weinreb et al., 2014) and impacts long-distance connectivity between the retina and the visual centers in the brain (discussed earlier). In retinitis pigmentosa, or rod-cone dystrophy (a group of inherited, mostly recessive diseases characterized by the onset of night blindness and gradual loss of peripheral vision, prevalence ∼1:3500 to 1:4,000) loss of rod photoreceptor cells triggers the late stage degeneration of cone photoreceptors even though specific mutation affects only rods but no cones (Kaplan et al., 2017). Once the photoreceptors die it causes remodeling of inner retinal neurons and followed by cell death of inner retinal cells (Singh et al., 2014). In addition, cone-rod dystrophies (inherited retinal dystrophies/maculopathies, prevalence 1:40,000) (Hamel, 2007) and Leber Congenital Amaurosis (very early-onset child blindness, usually autosomal-recessive, prevalence 1-2:100,000, source^3, 4^) add to the number of devastating blinding diseases affecting people and causing loss of life quality and partial loss of independence.

At present, there is no effective treatment available for most of these retinal disorders (except for wet AMD) despite most of the studies done on animal (mostly rodent) models to find new therapeutic options for retinal diseases. Rodent models can mimic only certain aspect of human retinal pathophysiology. They fail to reproduce the etiologic complexity of human RD diseases, including and especially some critically important characteristics of the primate retina like macula (Zeiss, 2010) [rodents don’t have macula (Volland et al., 2015a); cats and dogs have *area centralis* (Petersen-Jones, 1998; Mowat et al., 2008)] or trichromacy important for visual acuity in patients (Kostic and Arsenijevic, 2016), and do not always mimic the retinal disease phenotype (Slijkerman et al., 2015). Nevertheless, the neuroanatomical structure and connectivity of young retinal organoids growing in a dish is very similar to the *developing* human fetal retina, which is being explored as new way to study early stages of human retinal development (Meyer et al., 2009). However, all studies, where retinal organoids were cultured for prolonged period of time (6 months or longer), note the gradual changes in retinal organoids (specifically, gradual loss of RGCs and thinning of INL) (Wahlin et al., 2017; DiStefano et al., 2018; Brooks et al., 2019; Capowski et al., 2019; Nasonkin et al., 2019), thus substantially reducing the ability to model diseases and derive therapeutically meaningful results from drug screening efforts. Human retinal tissue in a dish has a real potential to be a great tool for drug screening, as well as disease modeling and source of transplantable 3D retina for RP and AMD after these critical shortcomings of retinal organoid technology are addressed. Scientific retinal community is keenly aware of the immense potential of human retinal organoid technology and the urgency of addressing these critical deficiencies in organoid technologies, preventing us to use it to the fullest extent for basic and translational research and regenerative medicine treatments. However, even now some remarkable success has been achieved with modeling treatments of some types of blindness (e.g., some ciliopathies) in retinal organoids, highlighting precise disease mechanisms and new potential therapies, which could be challenging to decipher and discover in cultured cells and time-consuming in animals (Schwarz et al., 2017).

In this review, we discuss the biology of retinal organoids and similarities with human retinal development, translational applications of retinal organoids in disease modeling (based on today’s technology state), cell or tissue replacement and discuss current major limitations of retinal organoid technology and how to overcome it. We provide a brief summary for each blinding disease (RP, AMD, glaucoma) to be aware of the current limitations as well as opportunities of retinal organoids as a tool for designing such models in a dish. We also use this summary throughout the text to discuss the key basic and translational research directions needed now to improve the retinal organoid models and technologies to enable faithful recapitulation of retinal biology, homeostasis and diseases in a dish for developing new drugs, delineating disease mechanisms and designing 3-Dimensional transplantable retina for replacement therapies.

We pay special attention to highlighting similarities and differences between human retinal organoids and human fetal and mature retinal tissue, and the impact of these similarities and differences on our ability to interrogate disease mechanisms, screen for drugs and use organoids for cell and tissue replacement therapies. Last, we present our opinion on how the technology will be developing in the next 3–7 years to focus on addressing the current limitations and urgent needs of biotech sector for developing therapies (drugs, biologics) using retinal organoids as a tool.

## Retinal Organoids for Basic Biology and Translational Studies

### Modeling Early Retinal Development

#### Early Cell Fate Decisions and Studying the Role of Morphogens

**FIGURE 1:**
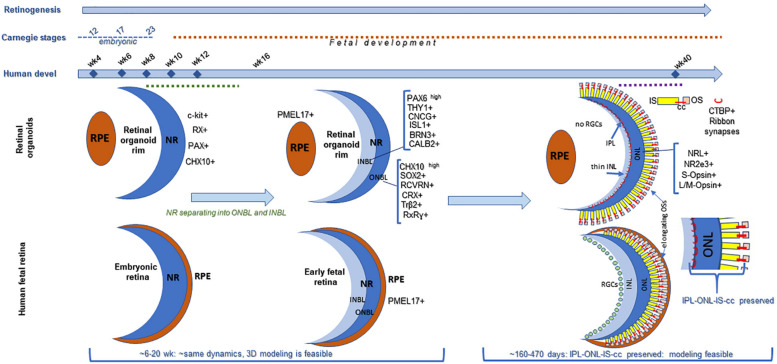
Comparing retinogenesis between human pluripotent stem cell derived retinal organoid growing in a dish and in human fetal retina. Both retinal organoids and fetal retina develop neural retina (NR), but only fetal retina (not organoids) develop continuous layer of RPE surrounding NR (Stage1). Both fetal retina and retinal organoids undergo lamination (Stage 2), and develop outer and inner neuroblast layers (ONBL, where progenitors, including photoreceptor progenitor and young photoreceptors localize, and INBL, where RGCs and 2^nd^ order neurons migrate). However, as tissue maturation proceeds in retinal organoids, the RGCs gradually die (no connectivity) and then a layer of 2^nd^ order neurons becomes progressively thinner. And, while cilia and inner segments in photoreceptors develop, outer segments never elongate full-length, compared to that in mature mammalian retina (Stage 3). In fetal retina (2^nd^−>3^rd^ trimester) photoreceptor layer undergoes maturation, cilia and inner segments are formed and elongation of outer segments takes place (which continues after birth). Organoids at stage3 preserve the features of immature human retina (IPL-ONL-IS-cc region, shown). This diagram is designed based on the following data: https://embryology.med.unsw.edu.au/embryology/index.php/Vision_-_Retina_Development, https://embryology.med.unsw.edu.au/embryology/index.php/Carnegie_Stage_Comparison, https:// embryology.med.unsw.edu.au/embryology/index.php/Carnegie_stage_table (O’Brien et al., 2004; Hendrickson et al., 2008; Jukic et al., 2013; Hendrickson, 2016; Hoshino et al., 2017).

Ongoing retinogenesis in 3D retinal tissue derived from hPSCs (ES and iPS) recapitulate early stages of human retinal development (Meyer et al., 2009; Volkner et al., 2016) (Figure 1). A number of very informative and well-designed retinal cell fate studies in young organoids were done by Gamm lab, which uncovered the instructive signaling of WNT and FGFs and decisions between NR and RPE fate (Capowski et al., 2016; Gamm et al., 2019). Developing retinal organoids (even without RPE) seem to be a good model for dissecting such major cell fate decisions, cell cycle, number of progenitors of each cell type and their initial organization in developing mammalian retina. Nevertheless, one should be mindful of some differences such as lack of RPE and lens and changes in the extrinsic factors and morphogen gradients caused by these differences (Dakubo et al., 2008; Smith et al., 2018). Though these signaling cues have some major consequences for translational research (e.g., BMP/TGFb signaling from ocular surface ectoderm through Smad4, also modulated HH signaling (Li et al., 2016), these are very early developmental processes (NR vs RPE), typically related to microphthalmia (Bharti et al., 2006; Manuel et al., 2008; Bharti et al., 2012) and marginally related to RD diseases.

#### Retinal Ganglion Cell Development

Retinal ganglion cell development takes place early in retinogenesis (Marquardt and Gruss, 2002), and young retinal organoids (∼6–15 weeks) derived by various methods carry RGCs (Singh et al., 2015), which are typically detected with antibodies to BRN3A, BRN3B, ISL-1, sometimes SNCG, HuC/D, neuronal-specific class III ß-Tubulin (TUJ-1 antibody) and/or Thy-1 (Barnstable and Drager, 1984; Huang et al., 2006). A number of labs pursuing RGC development successfully study RGC development, early stages of axonogenesis and axon guidance in retinal organoids (Fligor et al., 2018). In the absence of their natural target (visual centers in the brain (Cruz-Martin et al., 2014; Dhande and Huberman, 2014; Ray and Kay, 2015) RGC axons may even traverse through the retinal tissue (Singh et al., 2015) but eventually degenerate, together with RGC cell bodies (Wahlin et al., 2017; Capowski et al., 2019; Nasonkin et al., 2019). This is because RGCs need connectivity with the brain to receive flow of neurotrophins; RGC axotomy models and cases of anencephalic brain (both severing this vital to RGC connection) indicate rapid degeneration of RGCs in human fetal retina (Mo et al., 2002; Nakazawa et al., 2002; van Adel et al., 2005; Hendrickson et al., 2006). With the conceptualization and development of assembloid technologies co-culturing of retinal and brain organoids became feasible (Gopalakrishnan, 2019; Pacitti et al., 2019). This extends the developmental window for RGC studies in retinal organoid model which can now include studies interrogating mechanisms guiding RGC projections to brain (Kurimoto et al., 2010; de Lima et al., 2012; Erskine and Herrera, 2014; Crair and Mason, 2016; Benowitz et al., 2017; Laha et al., 2017).

#### INL & Outer Plexiform Synaptic Layer

Several long-term *in vitro* and *in vivo* studies investigated connectivity of second order neurons (rod bipolar neurons, typically stained with anti-PKCα antibody) and photoreceptors in human and mouse retinal organoids (Wahlin et al., 2017; Capowski et al., 2019) and *in vivo*, between graft or host-specific bipolar neurons and graft-specific photoreceptors (Assawachananont et al., 2014; Gonzalez-Cordero et al., 2017; Tu et al., 2018). The formation of CTBP2[+] horseshoe-like ribbon synapses (Singh et al., 2014; Singh et al., 2017) was documented by both IHC and electron microscopy in organoids cultured for 5–6 months or longer by several teams. In the *in vivo* studies, where the INL-specific organoid cells (including the bipolar neurons) continue to be lost, graft-specific photoreceptors were found in contact with (in some cases) graft-specific and (in some other cases) host-specific bipolar neurons (Assawachananont et al., 2014; Shirai et al., 2016; Tu et al., 2018). Collectively, these studies demonstrate that connectivity at the OPL level in organoids and in grafts is feasible, which lays foundation for tissue replacement work using hESC-3D retinal tissue from organoids as source of transplantable tissue. In anticipation of improved 3D human retinal models with functional RPE-photoreceptor niche and photoreceptor-second order neuron connectivity, it becomes important to focus on defining cone bipolar-photoreceptor connectivity and cone bipolar cell markers. The classical cone bipolar marker Recoverin (RCVRN) (Milam et al., 1993; Euler and Wassle, 1995) is also strongly expressed in CRX[+] photoreceptor progenitors (Singh et al., 2015) and α-RCVRN staining is the method of choice for defining photoreceptor layer in retinal organoids. Because of the major emphasis of translational retinal work on AMD (in addition to glaucoma) for building models of human macula and designing transplantable retina for patients with advanced AMD, delineating new reliable markers of cone bipolar cells for demonstrating cone bipolar-cone photoreceptor connectivity in organoids and in subretinal grafts may be critical for moving such modeling and transplantation work forward. Some excellent new markers were recently described (Shekhar et al., 2016).

#### Photoreceptors (Rod and Cone)

Photoreceptors (rod and cone) in retinal organoids are the key cell types in retinal organoids, which seem to remain organized in a uniform layer in long-term organoid cultures (Wahlin et al., 2017; Capowski et al., 2019; Nasonkin et al., 2019), and (in addition to RGCs) represent the primary drug-screening target for Big Pharma companies. Pioneering work has been done in human fetal retina by researchers like Drs. Anita Hendrickson, Tom Reh, Anand Swaroop and others to elucidated human retinal development with emphasis on photoreceptors (Abramov et al., 1982; O’Brien et al., 2004; Hendrickson et al., 2008; Hendrickson, 2016; Chao et al., 2017). A layer of photoreceptors with NRL[+] rods (Swaroop et al., 1992), OPN1SW[+] (S-cones) and TRß2[+] (Ng et al., 2001) M-cones robustly forms in organoids derived by multiple techniques, which highlights retinal organoids as a good model of human photoreceptor genesis and maturation in a 3D tissue in a dish. This enables to dissect the important of multiple small molecules, morphogens and canonical signaling pathways such as basic fibroblast growth factor (bFGF), docosahexaenoic acid, bone morphogenic protein (BMP), taurine, Retinoic Acid (RA), WNT (Wingless) (Murali et al., 2005; Pandit et al., 2015; Zhong et al., 2014; Capowski et al., 2016, 2019; Brooks et al., 2019; Gamm et al., 2019) important for photoreceptor development. Methods outlining mostly cone photoreceptor development from CRX[+] photoreceptor progenitors will be instrumental for modeling human macula in a dish as well as for designing transplantable 3D retinal grafts for treating patients with advanced AMD (Zhou et al., 2015).

## Signaling Pathways Involved in Human Retinal Development, Derivation of Retinal Organoids and Postmitotic Maintenance of Both Tissues

**FIGURE 2:**
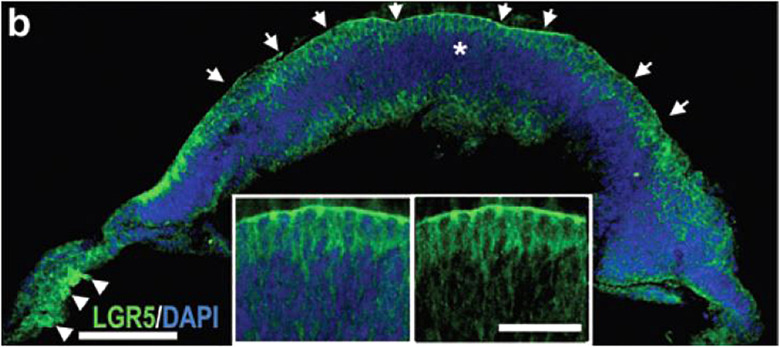
Presence of LGR5 in retinal organoids. Immunostaining of human pluripotent stem cell derived retinal organoid with anti-LGR5 antibody.

**FIGURE 3:**
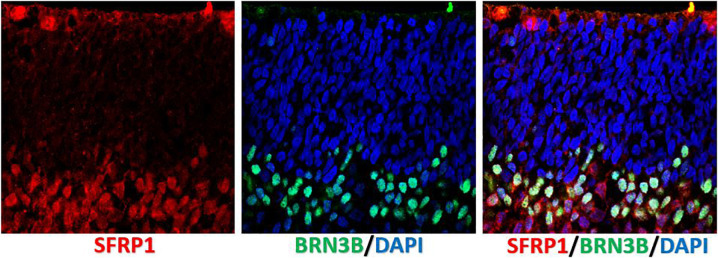
Localization of SFRP1 in human fetal retina. Immunostaining of human fetal retina (wk10) with anti-SFRP1 and anti-BRN3B antibody shows SFRP1 presence in the apical side of ONBL and distal side of INBL. Anti BRN3B colocalized with SFRP1 in the INBL.

Human retinal organoids recapitulate stages of human embryonic and early fetal retinal development (Meyer et al., 2009; Volkner et al., 2016; Gonzalez-Cordero et al., 2017) (Figure 1) and use the same pathways, active in developing human embryonic and early fetal retina for retinogenesis (Hoshino et al., 2017). The embryonic patterning and cell fate decisions in embryogenesis in general are regulated by very conserved developmental cues throughout the animal phyla (Perrimon et al., 2012). Human retinal development is not an exception and is shaped by the same cues and pathways (Heavner and Pevny, 2012). Some of these pathways also participate in maintaining retinal homeostasis. The importance of the *complex interplay* of these pathways in formation and further maturation of 3D human retinal tissue in a dish (organoids) only recently became a subject of thorough investigation (Hoshino et al., 2017). Understanding of this complexity will help with developing better retinal tissue-in-a-dish models with all retinal layers and functional RPE/photoreceptor niche for biopharmaceutical companies for drug screening (; Aasen and Vergara, 2020), and better retinal transplants for curing advanced retinal degenerative diseases (Assawachananont et al., 2014; Mandai et al., 2017a; McLelland et al., 2018; Shirai et al., 2016; Singh et al., 2019). Retina develops from the anterior portion of the neural tube through evagination of the optic vesicles from diencephalon, followed by invagination of those vesicles to form the optic cups carrying RPE and neural retina (NR) layers (consisting of the multipotential retinal progenitors) (collectively, “retina”) (Adler and Canto-Soler, 2007; Bharti et al., 2006; Fuhrmann et al., 2014). Invagination of each optic cup also leads to the formation of the optic stalk (the precursor of the optic nerve), which then becomes the optic nerve after the invagination of the stalk and closure of the choroid fissure (Remington, 2012; Forrester et al., 2016). MITF[+] RPE layer and CHX10 (same as VSX2[+]) NR layer carrying multipotential retinal progenitorsgive rise to the retinaand are collectively called “retina” (though some call NR “retina”, in contrast to RPE). Following their formation, RPE consistently remains as a single, layer, accumulates pigmentation and undergoes gradual maturation (Bharti et al., 2006, 2012), while NR undergoes a fascinating process of retinogenesis, where multipotential retinal progenitors sequentially acquire cell fate and form different types of retinal neurons and Muller glia (Livesey and Cepko, 2001; Marquardt and Gruss, 2002; Cayouette et al., 2006; Matsushima et al., 2011; Bassett and Wallace, 2012). Rod and cone photoreceptor cell fate acquisition and development is part of this retinogenesis process, and leads to the formation of the therapeutically valuable light-sensing outer nuclear layer (ONL) consisting of rods and cones (Swaroop et al., 2010; Ng et al., 2011). The default pathway in rod versus cone photoreceptor cell fate acquisition is cone PRs (specifically short-wave cones, S-cones) (Swaroop et al., 2010; Hunt and Peichl, 2014; Zhou et al., 2015). This pathway is promoted by blocking Bone Morphogenic Protein signaling (BMP), also WNT and TGFß signaling in culture (Zhou et al., 2015), and mutations of *NRL* or *NR2E3* genes *in vivo* (enhanced S-cone syndrome) (Mears et al., 2001; Sharon et al., 2003; Cheng et al., 2006; Littink et al., 2018). The expression of transcription factor Neural Leucine Zipper (*NRL*) at about week 10.5 of human fetal development defines rod photoreceptor cell fate (Swaroop et al., 1992; Mears et al., 2001; Hendrickson et al., 2008) and *NR2E3* (activated by *NRL*, at about week 11.7 in human fetal retinal development) (O’Brien et al., 2004; Cheng et al., 2006) further strengthens rod PR identity. Both of these transcription factors are expressed prominently in rod PRs in retinal organoids. *Waves of signaling mediated by WNT, FGF, Hedgehog, BMP/TGFb NOTCH, Retinoic acid (RA) and IGF-1 pathways through retina* shape retinal development from earlier stages toward the completion of retinogenesis (Yaron et al., 2006; Liu et al., 2006; Das et al., 2008; Fuhrmann, 2008; Fuhrmann, 2010; Fujimura, 2016; Mills and Goldman, 2017). Signaling via diffusible ligands is also present in postmitotic retina (Chen et al., 2015). These principles of development neurobiology combined with pluripotent stem cell technology are used for derivation of retinal organoids (good summary was provided in Dr. Sally Temple’s review Zhao et al., 2017). While some of these morphogen gradients seem to be present in retinal organoids (e.g., WNT pathway, judged by the presence of LGR5 and SFRP1 on the apical and basal side (Figures 2, 3) some other key gradients may be completely or partially absent due to lack of choroid, retinal vasculature and a continuous layer of RPE around the organoids. Exploring these signaling pathways in retinal organoids and comparing them to signaling in developing and postmitotic mammalian retina will improve the development of better 3D *in vitro* models of human retina, which may be particularly critical for drug development.

### WNT in Retina and Organoids

WNT is one of the most studied pathways in developing mammalian retina (Liu et al., 2006; Fuhrmann, 2008; Liu et al., 2010; Fujimura, 2016). Leucine rich repeat containing G protein-coupled receptor 5 (LGR5), a member of the G protein-coupled, 7-transmembrane receptor (GPCR) superfamily, is a receptor for R-spondins, and potentiates the canonical WNT signaling (de Lau et al., 2014). LGR5 is highly expressed in developing retinal organoids (300 fold) (Singh et al., 2015) and also present in developing human fetal retina (Chen et al., 2015). Formation of retinal organoids can be promoted by modulation of WNT pathway (Takata et al., 2017; Luo et al., 2018). WNT signaling remains important in postmitotic retina (Yao et al., 2016). WNT and Notch pathways acting together are known to regulate stem cell niches (birth, renewal and maintenance of multipotential stem cells, in short, stemness (Androutsellis-Theotokis et al., 2006; Clevers et al., 2014; Kessler et al., 2015) in tissues, including in the organoids (Kessler et al., 2015). Modulating these pathways in retinal organoids may be an interesting approach to study retinal regeneration and stemness (Jiang et al., 2020) to replenish cells lost in ageing, trauma or due to degenerative conditions.

### Sonic Hedgehog (SHH)

Sonic Hedgehog (SHH) and members of the Hedgehog (HH) family (Indian- and Desert Hedgehog, IHH and DHH) are well studied in developing vertebrate retina (Levine et al., 1997; Neumann and Nuesslein-Volhard, 2000; Nguyen and Arnheiter, 2000; Vogel-Hopker et al., 2000; Zhang and Yang, 2001a, b; Wang et al., 2002; Dakubo et al., 2003, 2008; Spence et al., 2004; Locker et al., 2006; Yu et al., 2006), including mammalian retina, more relevant to the biology of human retinal organoids, and provide very important paracrine signaling cues. There seem to be two major sources of HH signaling, one coming from RGCs (Wang et al., 2002), and another from choroid/RPE (from endothelial cells Dakubo et al., 2008, also from RPE Nakayama et al., 1998; Perron et al., 2003) impacting RPE (Zhang and Yang, 2001b; Burnett et al., 2017; May-Simera et al., 2018) and likely photoreceptors (Levine et al., 1997). HH signaling impacts retinal progenitor proliferation and cell fate determination (Wang et al., 2002; Sakagami et al., 2009). Cilia is needed for SHH signaling and is very important part of both types of cells forming the subretinal niche (photoreceptors Gilliam et al., 2012; Rachel et al., 2012a, b; Yildiz and Khanna, 2012; Wheway et al., 2014 and RPE May-Simera et al., 2018). Problems with cilia development, structure and functions result in ciliopathies, and many of them involve RD conditions because of importance of primary cilia for visual transduction, RPE-photoreceptor connectivity and outer segment function (Chen H.Y. et al., 2019). RPE cilia length had a noticeable change in Dnmt1 conditional mutants with short outer segment, hypoplastic apical RPE and retinal degeneration (Nasonkin et al., 2013). Pharmacological drugs were found, which promote apical RPE maturation in hiPSC-RPE and promote cilia formation. In relation to SHH, the integrity and shape of cilia impact SHH signaling efficacy, dependent on cholesterol (derived from OS membranes) (Myers et al., 2013; Bangs and Anderson, 2017; Garcia et al., 2018; Kinnebrew et al., 2019; Kong et al., 2019). Cilia is present in maturing hPSC-retinal organoids ∼5-6 month and older (demonstrated by our lab (Nasonkin et al., 2019) and others (Wahlin et al., 2017; Hallam et al., 2018; Capowski et al., 2019). With cholesterol provided via FBS (Yang et al., 2014) (critically needed for further growth of organoids) (Zhong et al., 2014) and photoreceptor cilia present in organoids ∼5–6 month old and older, SHH signaling is probably reconstituted in tissue culture. However, once the co-culture system between photoreceptor sheet in retinal organoids and the RPE (or RPE/choroid) sheet is created, this signaling will become closer to the one present in mammalian subretinal niche.

### FGF1 and 9

FGF1 and 9 are important for neural retina formation (less for cell fate specification Cai et al., 2010 except for RGCs Chen et al., 2013) and then for photoreceptor survival and maintenance (Fontaine et al., 1998; Qin et al., 2011; Hochmann et al., 2012), and were used to enhance NR cell fate in mouse models and *in vitro* in hPSC- > retinal differentiation systems (2D and 3D) at the expense of RPE (Pittack et al., 1997; Zhao et al., 2001; Horsford et al., 2005; Cai et al., 2010; Hambright et al., 2012; Gamm et al., 2019). Separately, some lower (reduced) level of basic fibroblast growth factor (bFGF) signaling (Moore et al., 2004) but not the complete abrogation of FGF signaling (Meyer et al., 2009) is needed for initial eye field specification. FGF morphogens are potentially a great tool to enrich for neural retina cell fate in organoids and 2D monolayer cultures. However, in view of the importance for developing better *in vitro* retinal models with RPE and NR growing together for studying and treating RD diseases, it seems that keeping the developmentally relevant balance of these factors, rather than completely abrogating RPE cell fate in developing retinal organoids, may be the right approach for developing better 3D retinal models. Nevertheless, investigating neuroprotective abilities of FGFs for promoting photoreceptor survival (Fontaine et al., 1998; Qin et al., 2011; Hochmann et al., 2012) seems a doable and therapeutically relevant approach in 6-12 - month old organoids, where the predominant surviving cell type is rod and cone photoreceptors.

### BMP and TGFβ Signaling

Contribution of BMP signaling is important in retinal development for determination of NR identity (Murali et al., 2005; Pandit et al., 2015) and originates from developing lens (Pandit et al., 2015), ocular surface ectoderm (Li et al., 2016) as well as retinal neurons (Close et al., 2005), RPE and vasculature (summarized in Ma et al., 2019). Activin A signaling through SMAD2/3 was found to increase number of photoreceptor precursors during retinal differentiation in 2D adherent monolayer (Lu et al., 2017). BMP/TGFb (and SHH) pathways modulation were used for derivation of organoids from human embryonic stem cells (hESCs) (Hallam et al., 2018; Kuwahara et al., 2019). Constitutive TGFβ signaling is needed in postmitotic retina (Ma et al., 2019). TGFβ (b1, b2, b3) are expressed by multiple developing and postmitotic retinal cell types including neurons, vasculature, RPE and microglia (Lutty et al., 1991; Lutty et al., 1993; Siegert et al., 2012; Close et al., 2005; Ma et al., 2019). Excessive TGFβ signaling causes epithelial to mesenchymal transition (EMT) in RPE and proliferative vitreoretinopathy (PVR) and fibrosis, while Cre-mediated deletion of *TGF*β in the whole eye and in vascular endothelium (but not RPE) caused choroidal neovascularization (CNV) (Schlecht et al., 2017). Both EMT- > PVR and CNV cause secondary changes in retina causing photoreceptor degeneration. These signaling are very relevant and important for developing vision restoration therapies (Kobayashi et al., 2019). However, in the absence of vasculature and subretinal niche in organoids we are so far limited in the ability to study them in the 3D retinal organoid model. A major source of BMPs and TGFs in retinal organoid culture is clearly delivered exogenously, with addition of 5–10% fetal bovine serum (FBS), and adding FBS is critical for maturation and growth (but not formation) of retinal organoids (Zhong et al., 2014). Modulating BMP-4 level in developing retinal organoids may help generate NR with RPE at the margin (ciliary margin-like zone, CMZ) (Kuwahara et al., 2015), which is a step in the right direction toward generating physiologically and therapeutically relevant 3D NR-RPE models (the subretinal niche).

*NOTCH pathway* is frequently mentioned when discussing retinal progenitor cell (RPC) proliferation/maintenance and specification, asymmetric cell division via Numb (where one RPC daughter acquires cell fate while another proceeds with symmetric cell division) (Shen et al., 2002; Ha et al., 2017) as well as regenerative cues in retina supporting retinal tissue regeneration (Mills and Goldman, 2017). Notch signaling in retina (which involves 4 receptors) works via paracrine ligands (Ha et al., 2017), with downstream signaling cascade involving RBP-J transcription factor (Riesenberg et al., 2009; Zheng et al., 2009) and Hes1/Hes5 (Yaron et al., 2006). Inactivation of RBP-J and modulation of Notch pathway with inhibitors (DAPT being the most well-known Ɣ-secretase/Notch pathway inhibitor) impacts the formation of NR, retinal lamination and may impact PR yield (Tomita et al., 1996; Yaron et al., 2006; Zheng et al., 2009), as well as determination of other cell types (Furukawa et al., 2000) depending on timing of Notch pathway inactivation during retinogenesis. Retinal cell types are born sequentially during retinogenesis (Livesey and Cepko, 2001; Marquardt and Gruss, 2002). Notch pathway promotes cell cycle progression in multipotential retinal progenitors (Yaron et al., 2006), while Notch suppression causes premature exit from a cell cycle, causing premature birth of later-developing cell types (Tomita et al., 1996). Therefore, it is clear that when Notch pathway is blocked earlier in retinal development (when e.g., cone PRs are developing), such modulation may increase cone PR yield (Yaron et al., 2006). This knowledge has been used productively for modulating the number of different cell types in human and mouse retinal organoids (Volkner et al., 2016). Because of the involvement of Notch1 in regeneration and major differences in species in the ability to regenerate retinal tissue we focused this paragraph mostly on reports outlining the role of Notch pathway in mammalian retina. Critically to retinal organoids (which usually do not have a sheet of RPE cells around PRs (Zhong et al., 2014). Notch signaling is active in RPE as well (providing signaling cues to nearby RPCs (Ha et al., 2017; Liu et al., 2013). Ablation of RPE in mouse development severely impacts retinal layer organization (lamination) (Raymond and Jackson, 1995). Likewise, lamination defects occur in two RBP-J -knockout mouse models (Riesenberg et al., 2009; Zheng et al., 2009) (summarized in Zheng et al., 2010), potentially pointing to the need of active Notch signaling (via RPE or diffusible Notch ligands) for organoid growth and contributing to retinal lamination in organoids. It is likely that serum provides some level of Notch ligands as it is needed for organoid growth (Zhong et al., 2014) and RPE-free lamination in retinal organoids has been reported (Capowski et al., 2019).

### Insulin-Like Growth Factor 1 (IGF-1)

Insulin-like growth factor 1 (IGF-1)is one of the pathways, which was instrumental for derivation of retinal progenitors from hESCs in 2D adherent monolayers (Lamba et al., 2006; Lamba et al., 2010; Hambright et al., 2012) and 3D retinal organoids (Mellough et al., 2015; Singh et al., 2015). IGF-1 is a very important extrinsic factor (morphogen) in developing retina and was shown to promote proliferation multipotential retinal progenitors (RPCs) via PI3K/Akt and MAPK/Erk pathways (Wang et al., 2018) and rod photoreceptor precursors in the fish (teleost) retina (Mack and Fernald, 1993). IGF-1 signaling in general regulates tissue growth and development in embryogenesis by supporting cell survival and cell cycle progression (Schlueter et al., 2007). The transition of rod photoreceptor precursors to mature post-mitotic rod photoreceptors is also promoted by IGF-1 (Yi et al., 2005; Pinzon-Guzman et al., 2011) and is regulated (at least partially) by phosphatidylinositide concentration and 3-phosphoinositide-dependent protein kinase-1 (PDPK-1) (Xing et al., 2018). IGF-1 receptor immunoreactivity is present in the ONBL (where photoreceptor progenitors reside in developing retina and retinal organoids) and in ONL of postmitotic mammalian retina (Greenlee et al., 2006). Another report mapped IGF-1 receptor (IGF-1R) as well as insulin receptor, IR, predominantly to photoreceptors and blood vessels, with very low level in other retinal cell types (Lofqvist et al., 2009). IGF-1 is also a component of FBS (Singh and Armstrong, 1997), and, given the importance of IGF-1 for photoreceptor maintenance [above and (Arroba et al., 2009)] as well as RPE maintenance (Zheng et al., 2018) and the need of growing organoids for serum (Zhong et al., 2014), IGF-1 will likely be included in serum-free (“defined media”) culturing methods of long-term organoid/RPE culture in the next few years. In addition, data from Igf-1^–/–^ mutant mice (a model of human neurosensory syndromic deafness/blindness) indicated the gradual loss of ERGs, retinal morphology and significant loss of connectivity between photoreceptors and their synaptic partners (loss of bassoon and synaptophysin) while only small changes in the INL (Rodriguez-de la Rosa et al., 2012), highlighting the importance of IHG-1 pathway for photoreceptors and the need for IGF-1 in long-term photoreceptor-RPE cultures.

### Retinoic Acid (RA)

Retinoic Acid (RA) is one of the best studied signaling pathways, active and important in many tissues during embryonic patterning and organogenesis (Rhinn and Dolle, 2012). Retinoic acid signaling is important at several stages of mammalian eye development, including promoting retinogenesis (Osakada et al., 2008, 2009; Cvekl and Wang, 2009; Lamba et al., 2010). Vitamin A and RA are indispensable for eye development and participate in several stages of eye and retina development (Matt et al., 2005; Cvekl and Wang, 2009). Early in development, *Raldh2* expression in the optic vesicle enables generation of RA signal needed for invagination of retina to form an optic cup (Mic et al., 2004). Retinoic Acid, a biologically active Vitamin A (retinol) derivative, serves as a ligand for nuclear receptors regulating gene expression (Duester, 2000; Duester, 2009) and regulates the expression of the key rod photoreceptor cell fate gene NRL (Khanna et al., 2006). However, the continuing presence of RA negatively impacts photoreceptor maturation (Nakano et al., 2012). Exposure to exogenous RA increased the number of rod and green cone photoreceptors and decreased the number of blue and UV cone opsin cells in zebrafish (Prabhudesai et al., 2005), suggesting RA as an instrumental factor in retinal organoid culture contributing to rod-cone photoreceptor development. The enzyme involved in RA synthesis (RALDH2) has been localized to RPE. Mimicking RA signaling in young retinal organoids for promoting photoreceptor development is now part of many protocols and can be done with addition of RA to differentiation medium (Zhong et al., 2014; Wahlin et al., 2017; Capowski et al., 2019). Retinol/RA was localized to photoreceptor outer segments in long-term retinal organoid cultures (Capowski et al., 2019) and light-responsive (mature) retinal organoids have been generated (Hallam et al., 2018). However, restoring the chemistry of the retinoid (visual) cycle (Kiser et al., 2014) and recycling of all-trans-retinol back to 11-cis retinal by RPE (canonical pathway for chromophore recycling) (Saari, 2000; Wang et al., 2009) for natural reintroduction into photoreceptor outer segments seems unattainable until the establishment of long-term co-culture between sheets of photoreceptors in retinal organoids and RPE and rebuilding of functional photoreceptor-RPE subretinal niche.

### Pigment Epithelium Derived Factor (PEDF, or SERPINF1)

Pigment epithelium derived factor (PEDF, or SERPINF1) signaling is an important paracrine and autocrine pathway in retina (Tombran-Tink et al., 1991; Malchiodi-Albedi et al., 1998; Tombran-Tink and Barnstable, 2003) for maintaining PR-RPE niche (Volpert et al., 2009; Akiyama et al., 2012), photoreceptor maturation (Jablonski et al., 2000; Akhtar et al., 2019) and survival (Comitato et al., 2018; Chen Y. et al., 2019). PEDF expression is a hallmark of RPE maturation and polarization (Strunnikova et al., 2010; Maruotti et al., 2015; McGill et al., 2017). High level of PEDF expression is produced by RPE differentiated from human pluripotent stem cells (Kanemura et al., 2013; Maruotti et al., 2015; Geng et al., 2017; McGill et al., 2017).

Pigment epithelium derived factor has a pleiotropic impact on many pathways, is considered a neuroprotective factor in retina and among other functions, may potentially have immunomodulatory function (Dawson et al., 1999; Gregerson et al., 2006; Ho et al., 2011; Chuderland et al., 2013; Nelius et al., 2013; Idelson et al., 2018). In view of increasing interest in co-culturing systems between photoreceptor sheets in retinal organoids and RPE sheets for recreating the subretinal niche, better 3D *in vitro* long-term retinal disease modeling as well as designing transplantable retinal patches, PEDF may become one of the important factors for establishing such cultures and maintaining homeostasis *in vitro* between the photoreceptors and RPE.

## 3D Retinal Tissue Models for Elucidating Disease Mechanisms & Drug Discovery

**FIGURE 4:**
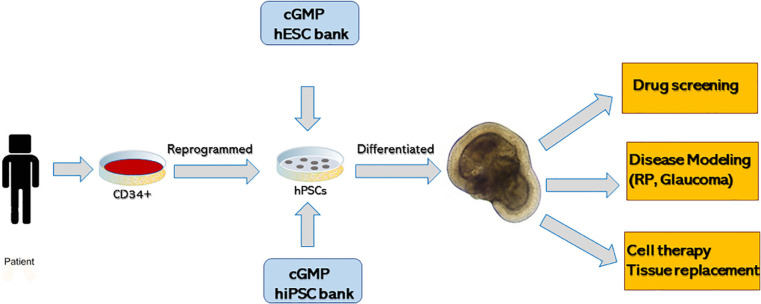
Summary of generating three-dimensional retinal tissue from human pluripotent stem cells and its application in disease modeling, drug screening and cell therapy or tissue replacement.

Though developmental biology questions were driving the discovery of retinal organoids, most work quickly shifted toward translational applications because of the unique ability to use organoids as a tool to design human retinal diseases in a dish (Figure 4). 3D-retinal organoids grown in a dish are developmentally, anatomically and physiologically similar to retinal tissue *in vivo.* Such ability has huge implication in disease modeling with organoids (Lancaster and Huch, 2019) although further improvements, particularly in formation of RPE and photoreceptor interaction and scalability, are definitely needed (Jin et al., 2011). Generation of human induced pluripotent stem cells (hiPSCs) from patients with retinal disease and further differentiating them to retinal organoid provide deep insight in understanding retinal diseases.

A number of studies have recently used retinal organoid for understanding retinal diseases (Jin et al., 2011), caused by *photoreceptor degeneration* (RP, AMD). Because most RP diseases are single-gene autosomal recessive, many RPs represent a very attractive target for organoid technologies for modeling and drug development.

Screening for small molecules ameliorating RD critically depends on the quality of model (retina-in a dish). For example, most RD diseases originate in the RPE-PR niche (either in PRs or/and RPE) and not having RPE-PR interaction in human retina-in-a-dish significantly impacts our ability to model these diseases (and screening for drugs preventing these RDs). Likewise, screening for drugs to ameliorate glaucoma (number one blinding disease) is so far challenging because RGCs degenerate in maturing retinal organoids (by ∼6 month in culture) (Capowski et al., 2019) due to the absence of the projection targets for RGCs (superior colliculus, lateral geniculate nucleus). However, a number of diseases focused on diseases originating at the level of photoreceptors can be studied and modeled. As one example, retinitis pigmentosa-39 (RP39) is caused by homozygous or compound mutations in *USH2A* gene, which encodes protein Usherin, required for photoreceptor (also hair cells in the cochlea) maintenance because of its role in cilia formation and function (Liu et al., 2007). In a study by Guo et al. the team reprogrammed cells from RP39 patient carrying (c.8559-2A > G/c.9127_9129delTCC) to iPSCs, generated mature retinal organoids from iPSC line with USH2A mutation and found significant defects in photoreceptor morphology with defective retinal progenitor cell development and retinal layer formation compared to control (Guo et al., 2019). Transcription profiling done on mutant retinal organoid revealed increase in apoptotic genes and abnormal gene expression compared to control. In another study iPSCs lines generated from three RPGR mutant patients [RP3 (Rozet et al., 2002), also RP15, cone-rod degeneration, X-linked (Mears et al., 2000)] were used for differentiation to retinal organoids (Deng et al., 2018). The team observed defects in photoreceptor morphology and localization, changes in transcriptional profiling and electrophysiological activity, in line with knowledge about the disease mechanisms. Interestingly, shortened cilium was found in patient iPSCs and RPE and photoreceptors in retinal organoids, derived from those iPSCs. Similarly, Megaw et al. (2017) showed that iPSCs-derived photoreceptors from *RPGR* mutation patients exhibited increased actin polymerization compared to the control, which was due to a disruption of cell signaling pathways regulating actin turnover via disruption of RPGR-Gelsolin interaction, which impacts Gelsolin activation (Megaw et al., 2017). Therefore, this study uncovered a disease mechanisms (loss of RPGR-mediated Gelsolin activation) using patient’s iPSC- derived retinal organoids as a tool, cheaper and faster than an animal model, and therefore identified a ***druggable pathway***, amenable for regulation with small molecules.

Study by Schwarz et al. (2017) identified interacting partners (Kif7 and Kif17) of the RP2 protein [GTP-ase activating protein (Veltel et al., 2008)] and observed reduced kinesin Kif7 (a conserved regulator of HH signaling) and Kif17 staining at photoreceptor cilia tips in iPSC-derived 3D optic cups from a patient with the X-linked *RP2* nonsense mutation c.519C > T (p. R120X) compared to control mutation-free organoids (Schwarz et al., 2017). The team was able to correct the PR2 defect by using ***translational read-through drugs***, collectively elucidating the disease mechanism/etiology and highlighting the potential therapeutic approach to treat the disease. Kif7 (together with another ciliary protein Kif17) is reported to play a role in stabilizing cilia tips yet prior to this study multiple other studies done in various models including zebrafish, *C. elegans* and mice failed to elucidate the precise mechanism of this blinding ciliopathy disorder [reviewed in Schwarz et al. (2017)]. Interestingly, the study from another group done in mice indicated that loss of RP2 protein is associated with cone but not rod photoreceptor defects and leads to abnormal extension of cone outer segments (Li et al., 2015).

In yet another study focused on iPSCs disease modeling of RP Masayo Takahashi’s team derived iPSCs from a patient with a RHO mutation, derived retinal organoids and demonstrated that photoreceptors in organoids recapitulate the disease phenotype and display signs of endoplasmic reticulum stress (Jin et al., 2012), typical feature in RHO models of RD (Kroeger et al., 2014).

### What RD Diseases Caused by PR Degeneration Can and Cannot Be Modeled so Far

From these examples it is evident that modeling of diseases originating within PR cell bodies (e.g., ER) and cilia may be modeled successfully and are not dependent/less dependent on the presence of RPE. The connecting cilium of photoreceptors is a very specialized structure providing stability for fragile and very compartmentalized photoreceptor OSs as well as enabling protein trafficking across the ISs between photoreceptor cell body and OSs (Pearring et al., 2013). Perturbing such protein traffic triggers photoreceptor cell death in many neurosensory ciliopathies, which involve not only vision but also hearing (Rachel et al., 2012a, b; Chen H.Y. et al., 2019). Photoreceptor disk formation is initiated at the level of cilia via specialized recently described mechanism of peripherin-dependent suppression of ciliary ectosome release (Salinas et al., 2017). The capture of photons and initiation of phototransduction takes place in the outer segments, which critically dependent on well-developed microvilli of apical RPE around them (Finnemann and Chang, 2008). RPE microvilli wrap around the tips of outer segments though do not reach the base of photoreceptor cilium (Marmor, 2013).

Interaction of photoreceptor OSs with microvilli of apical RPE is critical for phototransduction, retina-RPE adhesion, stability/homeostasis of photoreceptors and their OSs, and long-term sustaining of vision (Bazan, 2007; Goldberg et al., 2016; Kevany and Palczewski, 2010; Molday and Moritz, 2015; Palczewski, 2014; Wang and Kefalov, 2011). RPE supports photoreceptor function directly (via receptor-ligand mechanism) and indirectly (by secreting interphotoreceptor matrix, recycling 11-cis retinal for phototransduction etc.) (Bonilha et al., 2006; Finnemann and Chang, 2008; Sparrow et al., 2010). And, while models of photoransduction defects and perturbed OS renewal (all causing RD) (Lolley et al., 1994; Molday, 1998; Molday and Moritz, 2015; Petersen-Jones et al., 2018) clearly cannot be built *in vitro* until such 3D long-term co-culture is recreated in a dish, cilia formation and function seems to be recapitulated well enough in retinal organoids likely because it is not dependent directly on microvilli (Figure….) (Marmor, 2013). Therefore, when considering which RD diseases (discussed below) can be studied and modeled with retinal organoids, (some) ciliopathies may be an interesting and very important class of RD diseases (Chen H.Y. et al., 2019), which may be modeled even in the absence of RPE microvilli.

#### Retinal Ganglion Cells

Retinal ganglion cells are primarily affected in Glaucoma and other optic neuropathies and glaucoma, a leading cause of irreversible blindness in the United States and the world^5^. The main technological limitation of modeling glaucoma with retinal organoids is clearly the lack of the connecting partners of RGC neurons (visual centers in the brain). Yet, with the development of assembloids (retina-brain organoid co-cultures) technologies, this limitation seems to be only temporary.

The optic nerve originates in the retina and is formed by the axons of retinal ganglion cells (RGCs), the only type of retinal neurons, which requires long-distance connectivity (compared to other cell types, which use short distance connectivity: photoreceptor: bipolar neurons and INL neurons: RGCs). Modeling of RGC biology and disease in retinal organoids is challenged the need of RGCs to establish long-distance connectivity with visual centers in the brain to survive.**RGC viability** critically depends on their connectivity to visual cortex neurons, and such nerve fibers carry supportive (trophic) factors between RGCs and visual cortex neurons (Johnson et al., 2009). Damage to the optic nerve (e.g., the axotomy) can cause interruption or destruction of nerve cell connections and therefore, disrupt the flow of trophic factors leading to the gradual but steady loss of vision caused and RGC death. Restoration of trophic support (even partial) leads to preservation of RGCs (Mo et al., 2002; Nakazawa et al., 2002; van Adel et al., 2005). RGC layer will survive for months to years post injury as long as there is preservation of axonal connectivity between the RGC nerve fibers (forming the optic nerve) and the neurons of the visual cortex (Chang et al., 2006; Chang, 2013). One may find the retina in advanced degeneration stage (no photoreceptors and thin/degenerated INL) but with almost a normal RGC layer and optic nerve (Chang et al., 2002). However, it is feasible to study RGC development, organization and initial steps of axonal outgrowth to uncover factors promoting neurite elongation, guidance and target selection (Fligor et al., 2018). In the absence of their natural targets (visual centers in the brain) the RGC axons may grow randomly and even traverse the retina (Singh et al., 2015). With newly developed concept of retina-brain (“assembloids”) co-culturing methods (Gopalakrishnan, 2019; Pacitti et al., 2019) retinal organoids are becoming a very promising model of optic nerve regeneration, reconnection of retina to brain (Kurimoto et al., 2010; de Lima et al., 2012; Erskine and Herrera, 2014; Crair and Mason, 2016; Benowitz et al., 2017; Laha et al., 2017) and potentially glaucoma [when the chambers for ocular pressure mimicking may be designed for recreating intraocular pressure homeostasis (Acott et al., 2014; Wu et al., 2019)].

Collectively, these studies demonstrate that retinal organoids can be successfully differentiated from hiPSCs lines derived from retinal disease patients and used for delineating and modeling complex disease mechanisms, closely recapitulating the featured of RD diseases in patients. This in turn makes them reliable models for drug discovery.

#### Cell and Tissue Replacement Therapies for Retinal Degenerative Diseases

Before the arrival of retinal organoid technology, the aborted human fetal tissue (Radtke et al., 2008; Seiler and Aramant, 2012) and retinal progenitors derived from hPSCs (Banin et al., 2006; Lamba et al., 2006; Hambright et al., 2012) (embryonic and induced) were the two cell sources for transplantation. Human fetal tissue is a gift, with strong ethical restrictions and limited supply (NCSL, 2008; NIH, 2009; Finklea et al., 2015; Gerrelli et al., 2015; Wadman, 2015). A very promising and pioneering work on fetal retinal tissue transplantation has been done by Drs. Seiler, Aramant and Radtke (Radtke et al., 2002, 2004, 2008; Seiler et al., 2010; Lin et al., 2018). As discussed above, retinal organoids provide unprecedented way of approaching basic and translational aspects of human retinal biology for disease modeling, drug screening and also as source of retinal cells and retinal tissue for subretinal transplantation aimed at treating blindness.

Age related macular degeneration and RP/LCA are very good and tempting diseases for evaluating retinal sheet replacement strategies with retinal organoids (Assawachananont et al., 2014; Shirai et al., 2016; Mandai et al., 2017a; McLelland et al., 2018; Tu et al., 2018). Though both types of diseases are good targets for such therapy, AMD is not a purely genetic disease and etiology is not completely elucidated, while most RPs are recessive and can be avoided in the near future with advanced genetic testing and genetic counseling. There are at least 15 million people in the US affected by AMD, with at least 2 million having an advanced AMD stage. Macular patch approach, depending on organoid-derived photoreceptor sheet-RPE sheet coculture, is an attractive approach to bring vision to central retina. The size of human maculae is about 5mm in diameter (Kolb, 2005), and biological retinal patch ∼4 × 4 or 5 × 5 millimeters (mm) on a flat sheet of biomaterial carrier seems like a doable strategy (Ramsden et al., 2013; Mandai et al., 2017b; Kashani et al., 2018). It can be grafted to the back of patient’s eye to bring a layer of healthy and functional retinal tissue to replace patient’s own retina (too damaged/degenerated after the injury). This tissue is expected to reconnect (based on studies in mice) (Seiler et al., 2010, 2017) to patient’s RGCs and function as a bioprosthetic device similar to completely electronic chips currently approved for clinic (e.g., Argus II Stronks and Dagnelie, 2014). However, due to biological nature and much higher pixel density (which is expected to bring better vision Mathieson et al., 2012), where each individual light-capturing neuron [photoreceptor] of the patch is equal to a pixel, the biological retinal patch approach is expected to eventually supersede the electronic (neuroprosthetic) chip approach and to generate bioprosthetic retina capable of permanent integration into patient’s globe. A large piece of tissue from a hESC-derived retinal organoid carrying a layer of PRs and second order neurons provides the light sensors that can synaptically transmit visual information to patient’s RGCs, which persist even after all PRs are degenerated (Lin and Peng, 2013). Unlike electroprosthetic chips, a “bioprosthetic” implant based on hESC-derived retinal organoids enables long-lasting synaptic integration, and can be adjusted to carry more cones than rods (Mears et al., 2001) if the goal is to repair the macula. These technologies will be subject of intense studies in the next few years and will likely result in symbiosis of 2 approaches (biological and electronic) and neurobioprosthetic retinal implants, utilizing biomaterials and of course retinal organoids. Surgical technologies are already here to deliver such 3D constructs into the eye (Kashani et al., 2018).

CRISPR-Cas-9 gene correction in retinal organoids has been tested successfully in several human RD models (Deng et al., 2018; Huang et al., 2019; Lane et al., 2020) as well as *in vivo* in mice (with up to 45% efficiency of repair of dominant-negative Rho mutation to wild type allele in photoreceptors) (Li et al., 2018). CRISPR-Cas-9-based repair may be especially productive and needed for RP diseases, which are caused by dominant-negative mutations, and may potentially work together with retinal tissue replacement (discussed above).

## Current Limitations of Retinal Organoid Technology

The current key limitations of retinal organoids for modeling and treating RD diseases are the lack of vasculature, the lack of continuous layer of RPE around the organoids, gradual degeneration of RGC and then INL in mature organoid cultures, lack of the connecting partners for RGC axonal elongation (critical for glaucoma) and critically, lack of RPE-photoreceptor interaction (critical for dry AMD, RP and LCA). Here we discuss ongoing and future work needed to address these limitations.

### Absence of Vasculogenesis in Retinal Organoids

**FIGURE 5:**
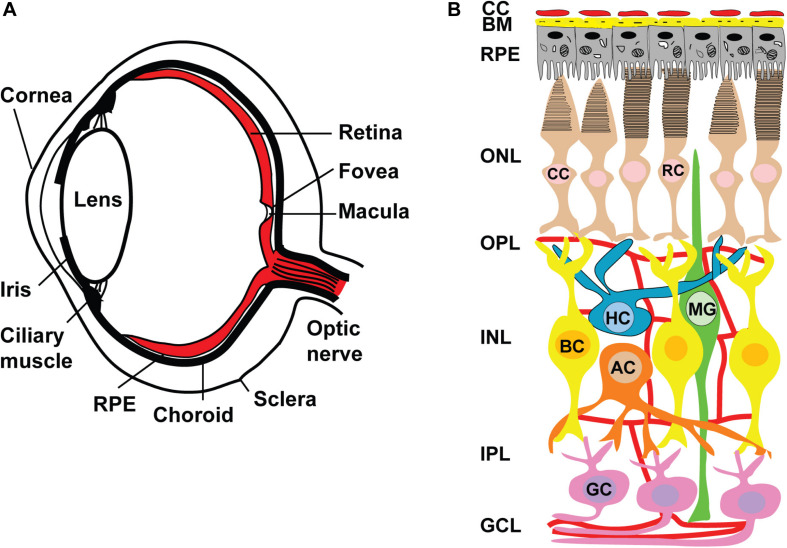
Anatomy of human eye and retinal circuits. **(A)** Schematic drawing of a cross-section through human eye. Light enters the eye through the cornea, passes through the pupil, lens and strikes the retina. Retina is the light-sensitive tissue lining the inner surface of the eye. Visual information from retina transmits to the brain through optic nerve fiber. In the middle of the retina small depression is called the fovea and is responsible for high resolution vision. Region surrounding the fovea is called as macula and are rich in only cones. Retinal pigment epithelium (RPE) is a pigmented layer and separates the choroidal blood supply from the photoreceptors. Choroid is a vascular layer of the eye. The sclera is a tough white sheath around the outside of the eyeball. **(B)** Schematic diagram of normal retina circuits. Mammalian retina consists of six major types of neuronal cells – rod (RC) and cone (CC) photoreceptors also horizontal (HC), bipolar (BC), amacrine (AC) and retinal ganglion cell (RGC). The Muller cell are the glial cells that span across the retina and their somata. RPE provides metabolic and transport functions essential for homeostasis of the neural retina. Bruch’s membrane (BM) is a highly specialized and multi-laminar structure in our retinas that forms the basis for mediating interactions between the retinal pigment epithelium and blood flow from the choroid. Choroidal capillaries (CC) are the blood capillaries present in choroid that supply oxygen and nourishment to the outer layer of the retina. Retinal blood vessels are present in OPL, IPL and RGC layers.

The retina is one of the most energy-demanding tissues, with high need for oxygen and nutrients (Warburg, 1928; Ng et al., 2015; Sun and Smith, 2018). Adult retina generates energy via aerobic glycolysis, in addition to oxidative phosphorylation, to compensate for such high demand (Ng et al., 2015) similar to cancer cells (Warburg et al., 1927; Vander Heiden et al., 2009). The oxygen and nutrient supply are delivered either from the choroid side (thus, RPE and photoreceptor layers are avascular and depend on choriocapillaris), or central retinal artery (which brings oxygen to RGC and INL; these 2 layers carry vascular capillaries). These energy demands are caused by energy-demanding phototransduction and related neurotransmitter demand caused by constant depolarization/repolarization (collectively: hyperactive neuronal activity) (Wong-Riley, 2010). However, the initial steps in retinal development lack vasculature (Hughes et al., 2000; McLeod et al., 2006; Hasegawa et al., 2008) (about week14), while the nutrition and oxygenation are delivered from choroid (beneath the RPE) and hyaloid (above the developing retina) (Ye et al., 2010) (Figure 5). This can be easily recreated in tissue culture incubator in smaller-size organoids, where penetration of oxygen and metabolites are not yet impacted much by organoid size. This indicates that initial stages of retinogenesis in a dish may be not impacted by lack of vasculature in organoids (i.e., neurovascular niche is not relevant at this stage, additionally confirming young organoids as a good model of early retinal development (Meyer et al., 2009)). Indeed, human fetal retina (∼Carnegie stage 23, day 56–60 and slightly older retina week 11–13) highly resemble human retinal organoids (∼week 10–12), as CHX10[+] NR with multipotential retinal progenitors is gradually separating into outer and inner neuroblast layers (ONBL and INBL). There are differences in the dynamics of retinal vasculature development in humans versus mice, though it is not clear if this is relevant to retinal organoid culture. However, in humans vascular development is complete before birth, while in mice and rats vascular development takes place postnatally [discussed in Sun and Smith (2018)]. There is ongoing productive work to recreate choroid-RPE border in a dish, which is a step in the right direction toward building a complete “retina in a dish” model (Jha and Bharti, 2015; Song and Bharti, 2016) from retinal organoids, RPE sheets and biomaterials. In general, organoid vascularization is a very active research niche at the moment (Grebenyuk and Ranga, 2019) since the initial stages (organoid formation) has been worked out. However, because of the clear differences between the laminated and heavily vascularized structure of human inner retina and spheroid “closed” and avascular structure of retinal organoids, where the oxygen and nutrition supply have difficulty penetrating into the INL/RGC layers (closer to the organoid core (Capowski et al., 2019)), it seems so far impossible to maintain the long-term dynamics of development in retinal organoids, shaped as a sphere. As a result, a number of labs, including ours, noted the almost exclusive survival of photoreceptors in the ONL and gradual demise of INL and RGCs in the organoid core (Wahlin et al., 2017; Capowski et al., 2019), highlighting older retinal organoids (maintained with current level of technology) as a questionable models for a number of RD conditions. Hypoxia inducible factor 1 (HIF-1) plays an important role in response of retina to oxygen level (hypoxic conditions), specifically alpha (HIF-1α) subunit, which becomes stable and translocates to the nucleus only in hypoxic conditions (Hughes et al., 2010). There is constitutive HIF-1a signaling reported in the normal rat and human retina suggesting an important physiological role (Hughes et al., 2010). The level of HIF-1a is high in both developing human fetal retina and young and mature (6-month-old) retinal organoids (based on RNA-Seq data sets from various publications). Because hypoxic conditions and active HIF-1a were reportedly noted as important for tissue regeneration (Nauta et al., 2014; Zhang et al., 2015; Heber-Katz, 2017; Lee et al., 2019), retinal organoids may be an interesting model for exploring retinal tissue (specifically photoreceptor) regeneration by modulating HIF-1a pathway, active in organoids.

## Lack of RPE Photoreceptor Interaction in Retinal Organoids

**FIGURE 6:**
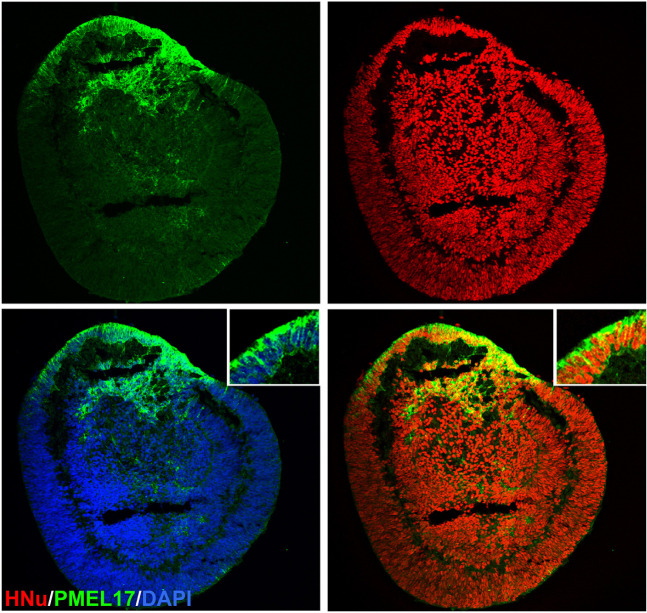
Localization of PMEL17 in the retinal organoid. Immunostaining the retinal organoid (day 70) with pigmented RPE marker PMEL17 show patches of retinal organoids were pigmented. HNu stains the human nuclei. The insets in panel a are high magnification of area marked with asterisk (*). DAPI counter stains nuclei.

**FIGURE 7:**
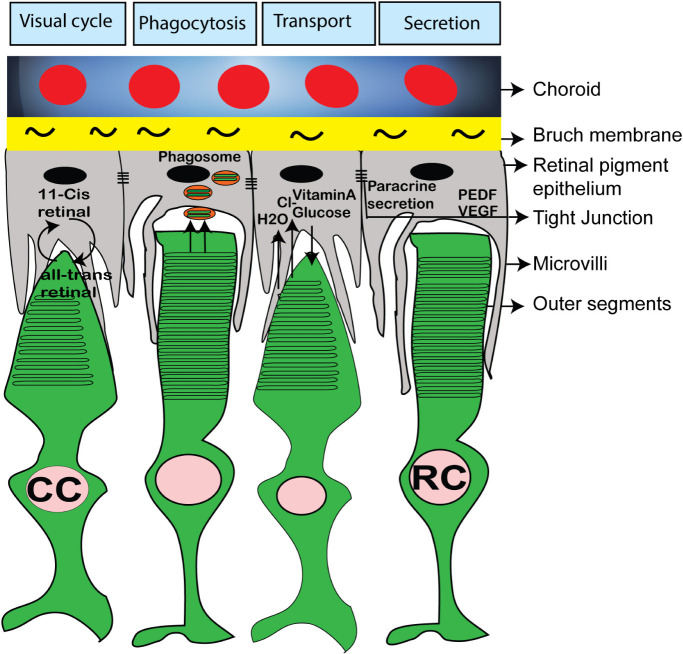
Schematic diagram showing important functions of RPE and its interaction with rod and cone photoreceptor outer segments. The RPE microvili interacts with photoreceptor OS and RPE cells are involved in visual cycle, phagocytosis of outer segments disc, nutrient uptake and paracrine secretion of PEDF, VEGF.

Retinal organoids do not have the continuous layer of RPE around the organoids (Figure 6). Achieving photoreceptor-RPE interaction and designing a functional subretinal niche in retinal organoids is an urgent goal critically needed for designing better models of human retina for drug development and for tissue replacement. As photoreceptors develop their specialized structure adapted for phototransduction, they elongate the inner and outer segments (ISs and OSs) into microvilli, elongating in sync on the apical RPE side (Figure 7). This elongation process takes place rapidly in developing mouse eye between approximately postnatal day 9.5 and 14.5 (Nasonkin et al., 2013), while in human developing retina the process starts in the 3^rd^ trimester and continues into infancy (Hendrickson et al., 2008). This indicates that to achieve outer segment elongation in human retinal organoid cultures one needs to wait approximately 24 weeks after the formation of retinal organoid, and 32 weeks or more to have OSs reach the maximum length, assuming that the organoid culture faithfully recapitulates human retinal development, and RPE-organoid co-culture system is established. Genetic ablation of RPE leads to complete loss of photoreceptor OSs (Longbottom et al., 2009), while hypoplastic changes in the apical RPE prevent OS elongation (Nasonkin et al., 2013), collectively pointing to the instructive and important role of RPE in outer segment elongation and maintenance. The interdigitation of OSs of photoreceptors and microvilli of apical RPE creates a stable NR-RPE border and a very specialized subretinal niche (absent or mostly absent in retinal organoids), where critical first steps of phototransduction take place in photoreceptor outer segments (Molday, 1998; Kefalov, 2012; Wang et al., 2009).

Photoreceptors have very compartmentalized structure adapted for phototransduction, which is supported by RPE microvilli (Molday and Moritz, 2015) (Figure 7). A number of extracellular (“interphotoreceptor”) cell matrix (ECM) proteins important and some critical for phototransduction and photoreceptor OS maintenance reside in the ECM matrix surrounding photoreceptor OSs and apical RPE microvilli (Bonilha et al., 2006; Ebrahimi et al., 2014; Ishikawa et al., 2015; Kelley et al., 2015; Salido and Ramamurthy, 2019). One of them is Interphotoreceptor Retinol-Binding Protein (IRBP), which plays a very important role of shuttling 11-cis retinal from RPE cells and bleached pigment (all-trans retinol) from photoreceptors to RPE (Figure 7) (Jin et al., 2009). IRBP message is abundant in human retinal organoids (based on published RNA-Seq data from various labs). A number of other interphotoreceptor proteins were highlighted in recent publications, some focused on studying these proteins specifically in retinal organoids (Felemban et al., 2018; Salido and Ramamurthy, 2019). There is a lot of interest now in these proteins among the teams, trying to use retinal organoids for modeling of retinal diseases and for cell replacement therapies (Dorgau et al., 2019; Guo et al., 2019). This interest is guided by the expectations that these “missing factors” (interphotoreceptor matrix proteins being some of them (Salido and Ramamurthy, 2019)) may help to build connectivity between RPE and photoreceptors in the organoids co-cultured with RPE *in vitro* to improve long-term culture of organoids and recapitulate the biology and structure of RPE-photoreceptor OS niche for disease modeling, drug screening and cell/tissue replacement therapies (Felemban et al., 2018; Achberger et al., 2019).

### Lack of Photoreceptor Disk Morphogenesis, Outer Segment Shedding, Phagocytosis in Retinal Organoids

These processes are fundamental to photoreceptor biology and phototransduction, and are critically missing in organoids (so far), thus reducing our ability to model many retinal degenerative diseases in a dish. Disk shedding occurs on the distal side of OSs facing the RPE, and these disks are phagocytosed by RPE, while disk morphogenesis takes place at the base of the OSs (next to the cilium) (Kolb, 1995; Molday and Moritz, 2015; Volland et al., 2015b). Up to 10% of OS discs are renewed daily (Young, 1967). The stack of rod photoreceptor OSs consists of over 1000 compact disk structures in adult retina (Molday and Moritz, 2015). However, the maximum number of disks we and others observed in the retinal organoids cultured for 6–8 months is limited to several disks, and these are not typically organized tightly in a stack (Wahlin et al., 2017; Nasonkin et al., 2019). However, some *in vivo* results reveal better organization of OSs and longer OSs in the long-term subretinal grafts (Shirai et al., 2016), all pointing toward the lumen of rosette-like photoreceptor aggregates in subretinal space (Shirai et al., 2016; Nasonkin et al., 2019). Naturally, there is no ongoing renewal and phagocytosis processes in long term retinal cell and retinal organoid cultures, but it has been expected for a long while that with developing photoreceptor-RPE co-culture systems faithfully recapitulating structure and function of the subretinal niche, OS elongation and photoreceptor-RPE biology can be reestablished (German et al., 2008; Di Lauro et al., 2016; DiStefano et al., 2018; Akhtar et al., 2019; Brooks et al., 2019; Capowski et al., 2019). As work on retinal repair rapidly progresses (Holmes, 2018a, b; Makin, 2019), these technologies will likely be developed in the next 3–5 years as a cheaper and robust model for drug screening and discovery, as well as platform for biomanufacturing 3D retinal tissue transplants to repair vision in advanced RD patients.

### The Visual Cycle

The visual cycle in mammalian retina (and lack of visual cycle in retinal organoids) Photoreceptors convert lights into an electrical signal that pass through the second and third layer of retinal neurons and conveys the information to the brain. Defect in RPE cell or photoreceptor cell impairs the visual function and causes retinal blinding diseases (Age related macular degeneration, Retinitis pigmentosa, Leber congenital amaurosis). The biochemistry of visual cycle has been worked out in seminal work of many laboratories (Hsu and Molday, 1993; Pugh and Lamb, 1993; Baehr and Palczewski, 2007; Luo et al., 2008; Arshavsky and Burns, 2012; Palczewski, 2014). Clearly, no similar Ca(2 +) or cGMP gradients (which are present in the subretinal niche) are present in developing retinal organoids though increased level of Na^+^, K^+^ and Ca^2+^ electric current are present in developing organoids (Singh et al., 2015). This could be one of many factors causing gradual degeneration of photoreceptors in long-term organoid cultures. It is expected that recreation of photoreceptor-RPE niche in a dish would make it possible to substantially increase the viability of photoreceptors in long-term *in vitro* cultures (Di Lauro et al., 2016; Achberger et al., 2019; Akhtar et al., 2019; Chen Y. et al., 2019), thus enabling disease modeling and drug screening of diseases, where the integrity of subretinal niche and photoreceptor-RPE structural and functional connectivity is of paramount importance for maintaining visual function.

### 11-cis Retinal

11-cis retinal is critical for visual process in the OSs and OSs do not have it if we don’t have RPE. In the RPE65 mutant dogs a lack of 11-*cis* retinal supply to the photoreceptors leads to very reduced function of both rods and cones (Gearhart et al., 2008) similar to that observed in RPE65-mutant mice (Redmond et al., 1998). RPE is critically important for maintaining visual function (Strauss, 2005; Bharti et al., 2006, 2011) and recycles retinal between photoreceptors and RPE (11-cis -all-trans retinal). Though 11-cis retinal (or, more stable for of it, 9-cis form of retinal (Fan et al., 2003)) can be provided in trans to enhance visual responses (Gearhart et al., 2010), this will not substitute for OS homeostasis, turnover and recycling in photoreceptor sheets from organoids and RPE sheets (as discussed above) unless the subretinal niche with close OS-microvilli interaction will be recreated in a dish. After all, though hESC- and hiPSC-derived RPE sheets can indeed phagocytose photoreceptor OSs (Carr et al., 2009; Idelson et al., 2009; Bharti et al., 2011) (one of many functions of RPE in subretinal niche (Mazzoni et al., 2014)), OSs start degenerate pretty quickly after retinal detachment unless physical reattachment takes place quickly, within a day or less (Fisher and Lewis, 2010). Retinal detachment negatively impacts the biological process of disk production and disk shedding. Though OS-specific proteins are synthesized, they start to localize to locations other than OSs: Opsin accumulates in the plasma membrane, Peripherin/rds appears in cytoplasmic vesicles. It was reported that proteins specific to cone OSs are more sensitive to OS damage, and after only one week of cone opsin mislocatization the expression of cone opsins is downregulated. It was noted that within 24 to 72 h after retinal detachment almost all rod and cone OSs display signs of OS degeneration: they are shorter, acquire abnormal morphology with disks not positioned as stacks (Fisher and Lewis, 2006; Wickham et al., 2013). These features of OS morphology are very similar to those observed by our lab and others in long-term cultures of human pluripotent stem cells (hPSC)-retinal organoids (Wahlin et al., 2017; Capowski et al., 2019; Nasonkin et al., 2019). severely shortened but yet visible OSs can persist for up to several weeks after retinal detachment (Fisher and Lewis, 2006; Wickham et al., 2013).

### Variability in Size, Shape, Photoreceptor Density, Lamination

Variability in size, shape, photoreceptor density, lamination determined by cell line-specific and protocol-specific differences (both derivation and maintenance) were described and documented (Capowski et al., 2019; Cowan et al., 2019; Mellough et al., 2019). However, it is feasible even with current technologies to culture and maintain organoids for longer than one year, as a proof-of-principle (Capowski et al., 2019). It quickly became evident that once the self-formation of retinal organoids is done (which can be achieved with a number of protocols maintaining and promoting the propensity of retina (the outpocketing of anterior neuroectoderm) to form, further maturation and long-term maintenance of retina-in-a-dish requires more sophisticated media providing more salts, anti-oxidants, a milieu of supporting paracrine factors (provided by serum or serum plus RPE conditioned medium), etc. (Zhong et al., 2014; Bardy et al., 2015; Singh et al., 2015; Di Lauro et al., 2016; Achberger et al., 2019; Akhtar et al., 2019; Capowski et al., 2019). These issues are mostly technical, and current biomanufacturing technologies allow generating large number of aggregates of a defined size (e.g., for large-scale drug discovery) if needed.

## Summary

Modeling early retinal development with hESC and hiPSC approaches (from eye field determination and before photoreceptor develop outer segments) seems the most straightforward and very productive way of using retinal organoids for basic and translational research (Meyer et al., 2009). With arrival of methods of co-culturing between brain and retinal organoids (assembloids (Gopalakrishnan, 2019)), engineering vascularization of organoids (Grebenyuk and Ranga, 2019) and developing pressurized chambers (for glaucoma studies, all discussed above) it will become feasible, and very soon, to use human retinal organoids for studying wet AMD and glaucoma and developing better drugs tested in models faithfully representing pathophysiology of these diseases.

### Conceptual Efforts

Conceptual efforts should be centered on better understanding of rebuilding PR-RPE niche with cells and layers of tissue (e.g., PR layer and RPE sheet), building models of AMD and glaucoma with functional PR-RPE niche, developing techniques for designing cone photoreceptor-only sheets with RPE for modeling of human macula, generating retina-brain organoids co-culture methods for studying and treating glaucoma, and (potentially) investigating vascularization of hESC-retina in a dish. This will open the door for multiple therapeutic/translational approaches (drug testing, photoreceptor transplantation, 3D retinal tissue transplantation). 3D human retinal tissue model on a chip is still unattainable but technologies are being developed to make this a reality in the next 5–7 years (McUsic et al., 2012; Gu et al., 2018; Achberger et al., 2019; Haderspeck et al., 2019; Masaeli et al., 2020). Big Pharma companies need this tool to do large-scale screening of drugs to suppress/ameliorate RD. This screening is not possible with mouse models (not human, expensive, cannot be scaled up) and not productive in cultured retinal cells. Most cells in primary retinal culture are represented by Muller glia after 2–3 several passages; immortalized cell lines have multiple changes in signaling pathways, lack of cell-cell connectivity and loss of many feature of retinal biology, critically needed to faithfully recapitulate pathology.

### Among Current Limitations

**FIGURE 8:**
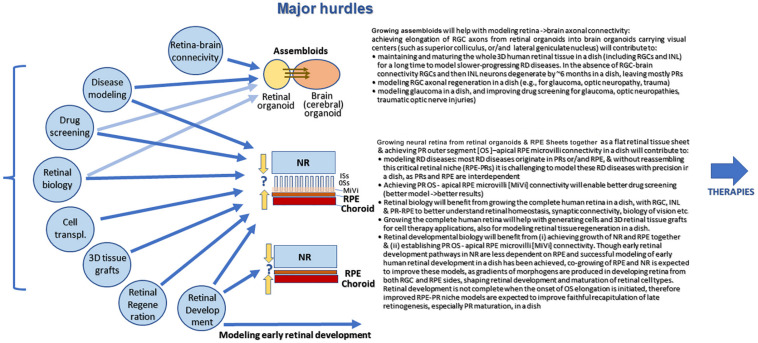
Major challenges of human pluripotent stem cell derived retinal organoid approach growing in dish. In retinal organoid there islack of RPE interaction with neural retina and lack of RPE interaction with choroid. Also, there is lack of retina brain connectivity. However, early retinal development can be studied using retinal organoid (cell fate acquisition). Once the advanced systems of co-culturing will be established (retinal-brain organoids =assembloids, photoreceptor sheets in organoids -RPE sheets, and vascularized organoids) one will be able to design better models of human retina for both drug screening and therapeutic applications.

Among current limitations one can list lack of functional maturation of PRs caused by lack of PR-RPE niche, premature degeneration, gradual loss of RGCs and second order neurons (Figure 8) and lack of connecting targets for RGCs to elongate and project. Difficulty for large-scale isolation of “good” retinal organoids and standardization of retinal organoid size and shape are also limitations yet it is expected that progress in biomanufacturing and biologic product development will soon be able to solve these hurdles.

### RPE-PR Interaction

RPE-PR interaction: (and lack of -in organoids) This hurdle is clearly biology-driven but the general expectation is that it could be circumvented with progress in technology, as organoid-biomaterial work progresses. Interestingly, even without RPE small stubby outer segments with rudimentary stacks of disks still grow, and in some cases even elongate, yet fail to develop organized stacks of outer segments, likely due to the absence of RPE-photoreceptor interaction. This interaction may be an inducing factor of elongation and most likely a stabilizing factor for disk formation. Equally interesting is the fact that there is hardly any data demonstrating the interaction between RPE and photoreceptors and elongation of outer segments in a dish, even in long-term organoid cultures.

The RPE layer is normally expected to polarize into apical and basal sides, and establish a network of microvilli on the apical side, interacting with photoreceptors and nurturing photoreceptor outer segments. Retinal organoids, however, normally carry patches of RPE on one side, thus directly exposing photoreceptors in the developing organoids to neural medium. Recreation of the critical retinal niche on the border between the apical RPE and photoreceptors, where many retinal disease mechanisms originate, is so far unattainable and is a focus of investigation in many labs. Interestingly, even without RPE small stubby outer segments with rudimentary stacks of disks still grow, and in some cases even elongate, yet fail to develop organized stacks of outer segments, likely due to the absence of RPE-photoreceptor interaction. This interaction may be an inducing factor of elongation and most likely a stabilizing factor for disk formation. Equally interesting is the fact that there is hardly any data demonstrating the interaction between RPE and photoreceptors and elongation of outer segments in a dish, even in long-term organoid cultures. Though the expectations are that the translational research in the near future may solve this hurdle (which is clearly biology-driven but could be clearly improved as organoid-biomaterial work progresses), so far the absence of this RPE-photoreceptor niche imposes clear limitations on both modeling/drug screening and transplantation approaches, especially for AMD/human macula work. Here we dissected the different retinal degenerative diseases and organoid technologies and present our thinking how and where retinal organoid technology can contribute the most to developing therapies even with a current limitation and absence of outer segments, elongating into the microvilli of RPE. Understanding how PR and RPE come together to rebuild functional subretinal niche is important not only for tissue transplantation and modeling of long-term RD disease in a dish, but also for promising cell therapy approaches, based on sorted photoreceptor transplantation (Singh et al., 2013; Gagliardi et al., 2018; Lakowski et al., 2018) (which is more feasible technically that grafting tissue (Seiler and Aramant, 2012) yet needs better understanding of biology to rebuild PR-RPE border with inner and outer segments). Photoreceptor (sorted CD73[+] cell suspension) transplantation so far has to depend on approaches circumventing this biological question like optogenetically engineered photoreceptors (Garita-Hernandez et al., 2019), where functional OSs, where light it converted to electricity are replaced by optogenetic constructs, mimicking OS function.

Now it is very interesting time in translational biology combining pluripotent stem cell technologies, biomaterials, 3D organoid and co-culture approaches in effort to model, rebuild in a dish and transplant the complex 3-dimensional tissue - the retina. This exciting time can be compared to the time when scientists were searching for elusive ways to dedifferentiate adult human cells back to pluripotent state. Developing 3D co-culture technology faithfully recapitulating the biology and physiology of the subretinal niche, together with organoid vascularization strategies, will open the new opportunities for designing better disease modeling in a dish to study the “late-onset” retinal diseases, which impact visual function at the lever of OS:RPE. In additional to biological breakthrough, this will be an ethical breakthrough, enabling us to avoid using animals excessively for studying development and treating diseases.

## Author Contributions

RS conceptualized the review, generated the data, and wrote the manuscript. IN conceptualized the review, processed the data, and wrote the manuscript.

## Conflict of Interest

Both authors were employed by the company Lineage Cell Therapeutics. The authors declare that the research was conducted in the absence of any commercial or financial relationships that could be construed as a potential conflict of interest.

## Footnotes

1 https://www.nei.nih.gov/learn-about-eye-health/resources-for-health-educators/eye-health-data-and-statistics

2 https://www.brightfocus.org/macular/article/age-related-macular-facts-figures

3 https://rarediseases.org/rare-diseases/leber-congenital-amaurosis/

4 https://ghr.nlm.nih.gov/condition/leber-congenital-amaurosis

5 www.glaucoma.org
